# Ampicillin treatment in persister cell studies may cause non-physiological artifacts

**DOI:** 10.15698/mic2025.03.845

**Published:** 2025-03-20

**Authors:** Michel Fasnacht, Hena Comic, Isabella Moll

**Affiliations:** 1 Max Perutz Labs, Vienna Biocenter Campus (VBC), Dr.-Bohr-Gasse 9 / Vienna Biocenter 5, 1030, Vienna, Austria.; 2 University of Vienna, Max Perutz Labs, Department of Microbiology, Immunobiology and Genetics, Dr.-Bohr-Gasse 9 / Vienna Biocenter 5, 1030, Vienna, Austria.

**Keywords:** persister cells, antibiotic treatment, ampicillin, ribosomal proteins, extraribosomal functions of r-proteins

## Abstract

Persister cells are a clinically relevant sub-population of an isogenic bacterial culture that is tolerant to bactericidal antibiotics. With the aim to investigate the ribosomal protein content of persister cells, we employed the bacteriolytic properties of ampicillin to separate persister from sensitive cells. Thereby, we observed processing of several ribosomal proteins. Promisingly, we detected a variant of the large subunit protein uL2 that lacks the last 59 amino acids from its C-terminus (tL2) and which previously has been described as an inhibitor of DNA replication *in vitro*. Considering the increasing number of moonlighting functions described for ribosomal proteins, we investigated a potential regulatory role of tL2 in persister cells after ampicillin treatment. In contrast to our assumption, our findings show that the generation of tL2 after ampicillin treatment must be attributed to proteolysis upon cell lysis. Ultimately, no tL2 was detected intracellularly of purified persister cells isolated by an improved protocol employing proteinase K treatment. We therefore exclude the possibility of tL2 regulating DNA replication in ampicillin tolerant *E. coli* cells. Nevertheless, this study clearly highlights the necessity of further purification steps in addition to ampicillin treatment for the study of persister cells and invites for the careful re-examination of previously published results.

## Abbreviations

r-protein - ribosomal protein, 

rRNA - ribosomal RNA. 

## INTRODUCTION

Persister cells are a sub-population of an isogenic bacterial culture that is tolerant to bactericidal antibiotics 1,2. Unlike antibiotic resistant bacteria, persister cells do not carry a specific resistance gene to combat the antibiotic and are thus unable to replicate in the presence of the drug. Instead, antibiotic persistence is typically characterized by a biphasic killing curve 1,2. Importantly, when the antibiotic treatment is stopped, persister cells are able to form a new, but still antibiotic sensitive culture. While the clinical relevance of persister cells has become increasingly clear 2,3,4,5,6,7,8,9, a unifying understanding of the underlying mechanisms of persister cell formations remains elusive and debated 10,11. Two broader models of persister cell formation have been described: the spontaneous and the triggered model. Spontaneous persistence is rare and occurs stochastically during steady-state exponential growth without any external trigger. In *Escherichia coli*, roughly 0.0001% of exponentially growing cells are estimated to be in a persister state 12. In contrast, the more common triggered persistence occurs as a response to a previous stress signal such as starvation or antimicrobial drugs and can drastically increase the number of persisters in a population (reviewed in 2,3). Independent of the type of formation, persister cells are generally thought to be dormant with a suppressed metabolism and reduced translational activity 3, but examples challenging the importance of dormancy have been described 13,14,15,16. Recent work employing single-cell RNA sequencing of persister cells proposed translational deficiency as an important feature of bacterial persistence 17. This proposal is in line with several previous observations. First, deacylated tRNAs accumulation, lower levels of translation, and pre-treatment with antibiotics targeting the translational machinery have been shown to increase tolerance to bactericidal antibiotics 18,19,20. Second, low levels of translational activity still observed in persister cells resulted in the synthesis of ribosomal proteins (r-proteins) and ribosome-associated proteins like the ribosome hibernation factor RaiA 21. Third, degradation of ribosomes in the stationary phase 22,23 correlates with an increase of persister cell count 24. Lastly, *E. coli* ribosomes have previously been found to be disassembled and degraded in persister cells 25 and moreover, ribosome content has been shown to dictate persister cell waking 26, highlighting the role of the ribosome as a regulatory hub of persistence.

The typical *E. coli* ribosome generally consists of three ribosomal RNA (rRNA) molecules and 54 r-proteins. While the core functions of the translation process are catalyzed by different nucleotides of the 23S and 16S rRNA, r-proteins still play a crucial role in ribosome biogenesis, stability, and functionality. Hence, many of the r-proteins are strongly conserved among all three domains of life 27. Recent research has highlighted increasing numbers of r-proteins that serve a secondary, moonlighting function outside of the ribosome (reviewed in 28,29). One important extraribosomal function of r-proteins includes the negative feedback regulation of translation initiation on operons encoding for different r-proteins to ensure the stoichiometric homeostasis between rRNAs and r-proteins. For example, uL4 inhibits both the transcription and the translation of the *S10* operon, which includes the *rplD* gene containing the genetic information for uL4 itself 30. In total, the *S10* operon comprises genes for eleven r-proteins, including *rplB* encoding the 50S subunit protein uL2 (Figure S1A). uL2 is highly conserved 31. In the assembled ribosome, uL2 contributes to the intersubunit bridge B7b via its globular, solvent-accessible domain 32, while its C-terminus extends towards the peptidyl transferase center (Figure S2) and plays a crucial role in efficient rRNA binding 33. Besides, uL2 has been shown to affect tRNA binding, and mutations in its C-terminus impacted the efficiency of the peptidyl transfer reaction 34,35. In addition, several extraribosomal activities have been proposed for uL2. First, *in vitro* and *in vivo* analyses suggested uL2 to interact with the α subunit of the RNA polymerase and thereby stimulate transcription from the ribosomal promoter *rrnD* P1 36. Shortly after, Chodavarapu *et al*. serendipitously found a truncated variant of uL2 (tL2) that lacks the last 59 amino acids from the C-terminus in cell lysates from exponentially growing *E. coli* cells 37. Using a series of *in vitro* experiments, the authors obtained evidence that both full-length uL2 and truncated tL2 were able to interact with the N-terminus of DnaA 37, the initiator of DNA replication 38. Based on their results, the authors concluded that tL2 might serve to fine-tune *E. coli* chromosome replication by inhibiting DnaA activity. However, Chodavarapu *et al*. did not observe a negative effect on growth rates upon artificial overproduction of tL2 *in vivo* and failed to identify any conditions leading to upregulated tL2 levels 37.

Considering the low translation efficiency in persister cells 17, the increasing number of moonlighting functions described for r-proteins 28,29, and the observed disassembly of ribosomes in persister cells 25, we aimed to investigate whether released r-proteins contribute to the survival and/or recovery of persister cells. As the bacteriolytic properties of ampicillin have routinely been used to separate non-tolerant from persister cells 24,26,39,40,41,42,43,44,45,46, in this study, we treated *E. coli* cultures with high doses of ampicillin to scrutinize the r-protein content in surviving persister cells. Surprisingly, we found several truncated r-proteins after the treatment, including the described tL2 protein 37. Further analysis revealed that lower, non-lytic concentrations of ampicillin as well as treatment with other non-lytic antibiotics did not lead to the production of tL2. To scrutinize potential regulatory roles of tL2 for the survival of high doses of ampicillin, we further purified persister cells after the antibiotic treatment. However, microscopy imaging of persister cells isolated according to previously established protocols 39 revealed that the removal of cell debris was incomplete. We therefore employed an additional protease treatment step to remove extracellular proteins and cell debris. While subsequent fluorescent microscopy imaging confirmed the successful removal of debris aggregates, no tL2 was detected intracellularly of the purified intact persister cells. Taken together, our results indicate that the generation of tL2 is an artifact of cell lysis and do not point to a physiological function of tL2 under the tested conditions. Furthermore, we found for several other r-proteins that initially showed processing upon ampicillin treatment that the corresponding cleavage products were absent from the intact persister cells, supporting the notion that this proteolytic cleavage upon cell lysis is a more widespread phenomenon. Our work therefore highlights the importance of employing additional purification steps beyond sequential centrifugation for the isolation and investigation of persister cells and invites for the careful re-examination of previously published results.

## RESULTS

### Ampicillin treatment generates r-protein truncations

**Figure 1:**
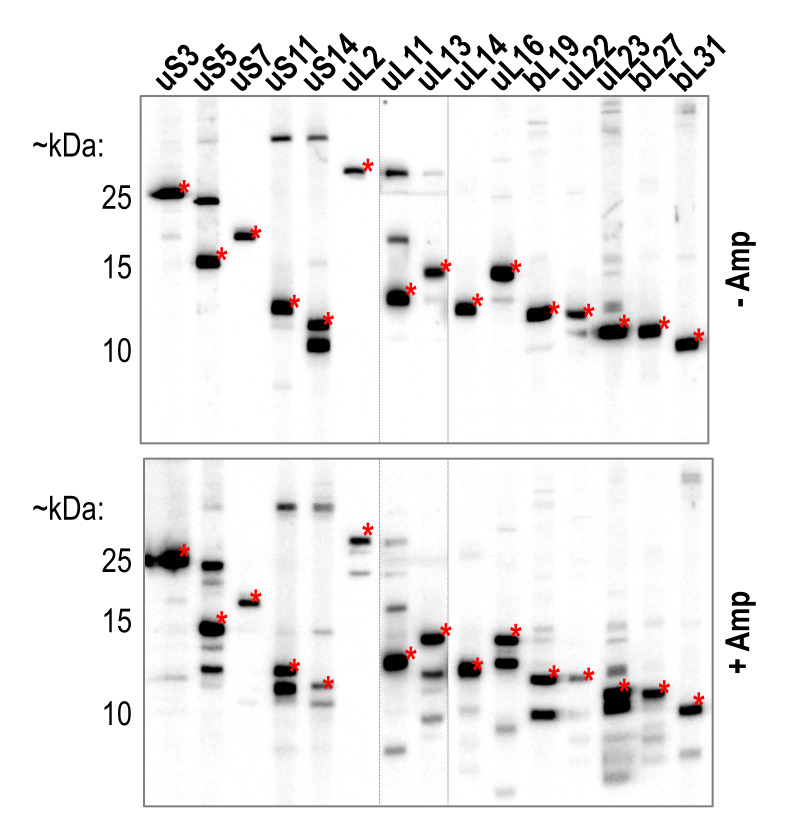
FIGURE 1: Most of the tested r-proteins are processed upon ampicillin treatment. Western blot analysis using a Mini-PROTEAN II multiscreen apparatus from Bio-Rad. Samples were harvested from wildtype *E. coli* strain BW25113 before (-Amp) and 2 hours after treatment of exponentially growing cells with 100 µg/mL ampicillin (+Amp). Different primary antibodies specific for the above indicated r-proteins were used in each slot. Based on the expected size, signals corresponding to the full-length r-protein were marked by a red asterisk. Due to the significantly lower signal intensity achieved with antibodies against uL11 and uL13, a longer exposure time image is superimposed at the corresponding slots.

**Figure 2:**
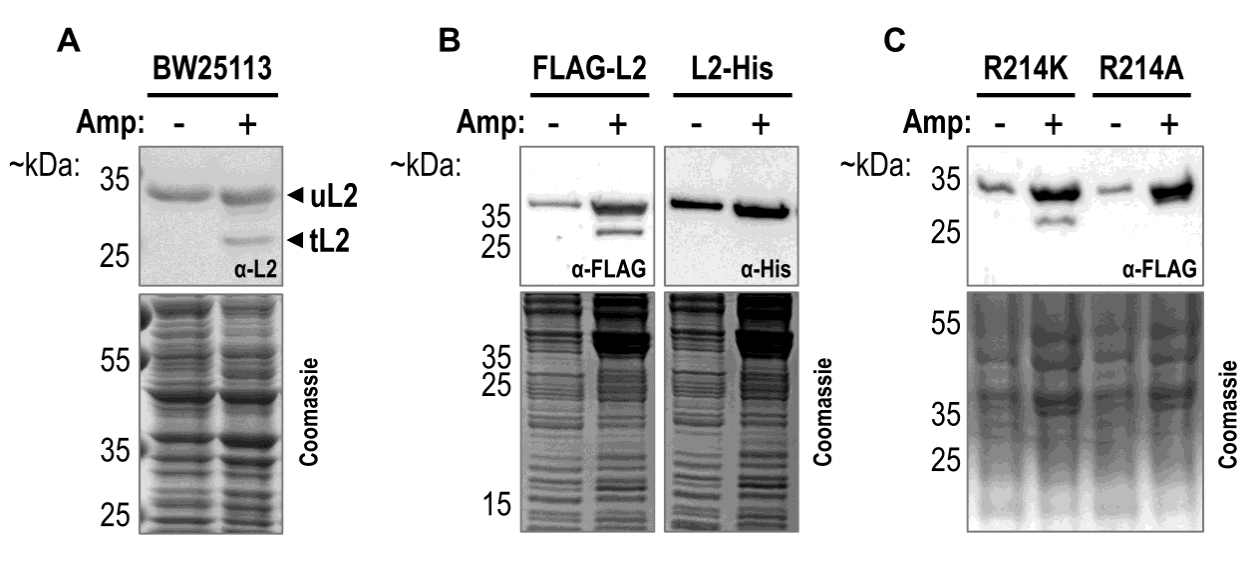
FIGURE 2: A truncated variant of uL2 lacking 59 amino acids at the C-terminus is produced upon ampicillin treatment. Western blot analysis of tagged and untagged forms of ribosomal protein uL2 before and after treatment of exponentially growing *E. coli* cells with 100 µg/mL ampicillin (Amp) for 2 hours. Primary antibodies used are indicated inside of the western blot boxes and Coomassie gels were used as loading controls. **(A)** Ampicillin treatment of wildtype *E. coli* strain BW25113 reveals that a shortened variant of uL2 (tL2) is produced after the addition of the antibiotic. **(B)** Truncation of uL2 was only observed when wildtype uL2 was ectopically synthesized from the plasmid pZS*33-lacZa-FLAG-rplB resulting in an N-terminal FLAG-tagged variant (FLAG-L2) and not when harboring a C-terminal His-tag (L2-His) encoded by plasmid pZS*33-lacZa-rplB-His. **(C)** Truncation of FLAG-L2 was still detected when the codon for arginine 214 on the plasmid pZS*33-lacZa-FLAG-rplB was mutated to a lysine codon (R214K) but not to an alanine (R214A) codon.

To investigate the r-protein makeup of beta-lactam antibiotic induced persister cells, we treated exponentially growing *E. coli* BW25113 cells with 100 µg/mL ampicillin for two hours to lyse non-persisters and directly harvested the remaining persisters by centrifugation for western blot analysis as previously described (39, see also material and methods). Specifically, we analyzed several r-proteins previously found to differ quantitatively between untreated cells and persister cells isolated after ampicillin treatment 25. While signals corresponding to the full-length r-proteins were still detected upon ampicillin treatment, we furthermore observed processing of most of the tested r-proteins (**Figure 1**). Most promisingly, we discovered two different isoforms of the large subunit protein uL2 (**Figure 2A**). As described above, Chodavarapu *et al*. previously identified a C-terminally truncated form of uL2 with a potential regulatory function in DNA replication 37. In their study, uL2 was cleaved after arginine 214, leading the authors to speculate that a trypsin-like protease, cleaving proteins after arginine and lysine residues, might be responsible for the processing of uL2. To confirm whether our observed shortened form of uL2 corresponds to the tL2 identified by Chodavarapu *et al*., we employed ectopic expression of *rplB *equipped with a 5´-terminal FLAG-tag or a 3´-terminal His-tag sequence. As shown in **Figure 2B**, we detected the shortened variant of uL2 upon ampicillin treatment solely with antibodies specific for the N-terminal FLAG-tag, confirming that the observed truncation occurs at the C-terminus of uL2. Furthermore, we replaced the codon for arginine 214 of *rplB *either by a lysine (R214K) or alanine (R214A) codon. As shown in **Figure 2C**, loss of the shorter uL2 is only observed with the R214A mutation, indicative for processing of uL2 by a trypsine-like protease at this position. We therefore concluded that the addition of high doses of ampicillin to exponentially growing *E. coli* cells engenders the tL2 variant previously described by Chodavarapu *et al*. 37.

### tL2 production occurs only after prolonged ampicillin treatment

**Figure 3:**
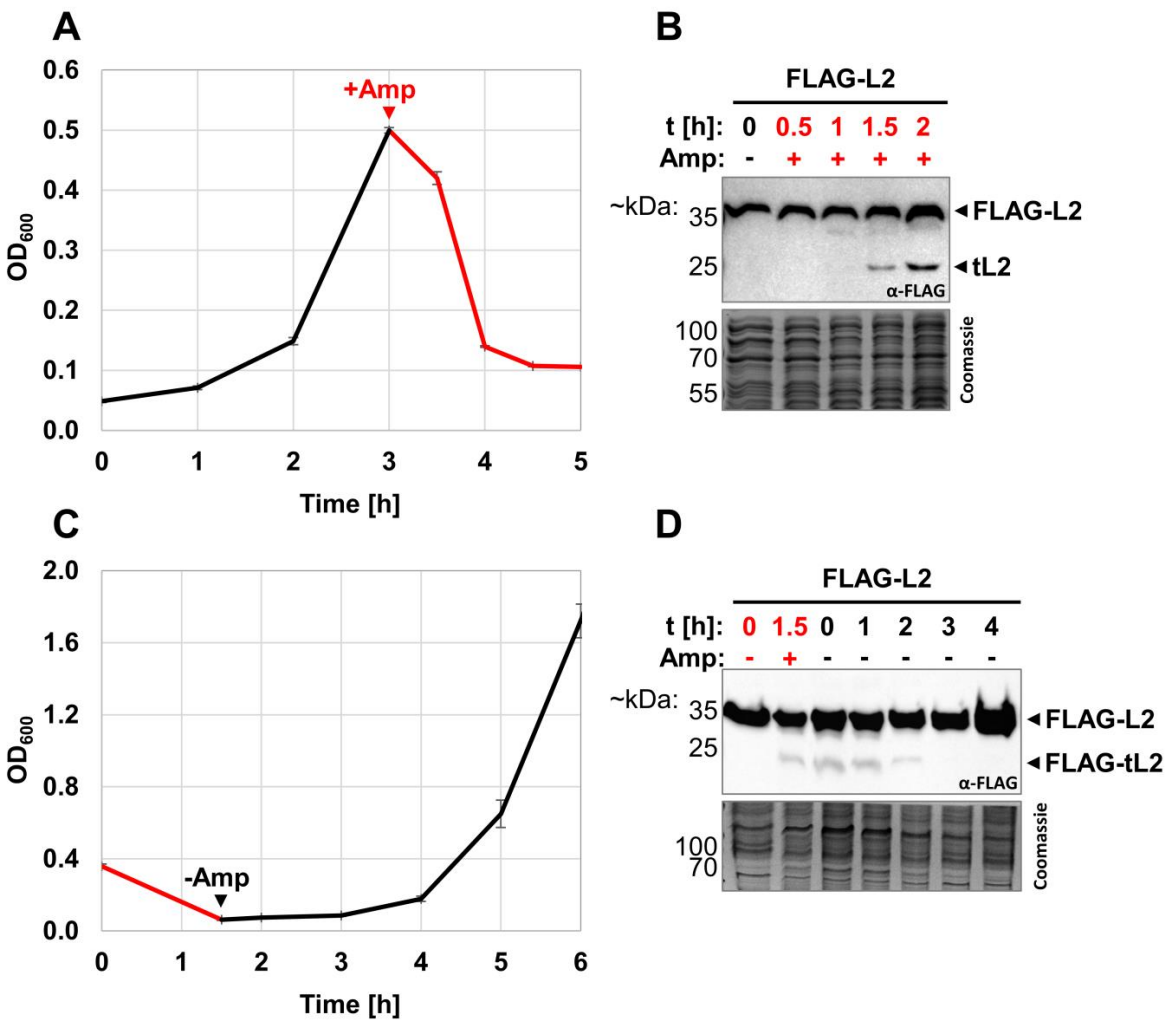
FIGURE 3: tL2 is slowly generated in response to ampicillin treatment and disappears upon recovery. **(A)** Growth of *E. coli* strain BW25113 harboring plasmid pZS*33-lacZa-FLAG-rplB in LB medium was monitored by measuring optical density at 600 nm before (black) and after (red) treatment with 100 µg/mL ampicillin (n=3). Samples were taken for western blot analysis in **(B)** before the addition of the antibiotic at OD_600 _= 0.5 (t=0h) and at the indicated time points afterwards. **(C)** Growth of BW25113 pZS*33-lacZa-FLAG-rplB cells treated with 100 µg/mL ampicillin was monitored by measuring optical density at 600 nm before (red) and after (black) wash and resuspension in fresh LB without ampicillin added. Samples were taken for western blot analysis in **(D)** before (t=0h, red) and 1.5h after the addition of the antibiotic, as well as immediately after resuspension in fresh LB (t=0h, black) and at the indicated time points thereafter.

Increased numbers of dormant, non-dividing cells have previously been shown to increase the survival of a bacterial population upon antibiotic treatment 24,26,44,47,48,49. Thus, our finding tempted us to speculate that inhibition of DNA replication by tL2 represents a previously unrecognized mechanism for persister cell formation. We therefore set out to further characterize the kinetics of tL2 production upon ampicillin treatment. As previously, ampicillin was added to exponentially growing cells and samples were taken for western blot analysis over the course of two hours (**Figure 3A **and **B)**. In contrast to the immediately observed growth arrest and widespread cell lysis upon treatment with high doses of ampicillin (**Figure 3A**), we detected protein tL2 only approximately 90 minutes after the addition of the antibiotic **(Figure 3B)**. As discussed earlier, a hallmark of persister cells is the ability to resume growth normally after the removal of the stress. If tL2 inhibits DNA replication to induce a dormant state during ampicillin treatment, we consequently expected a loss of the tL2 signal when the stress is removed and the surviving persister cells are recovering. We indeed observed a good correlation between the presence of tL2 and the recovery of growth upon exchange of the ampicillin containing medium to fresh LB as shown in **Figure 3C** and **D**.

### tL2 is only detected under lytic conditions

**Figure 4:**
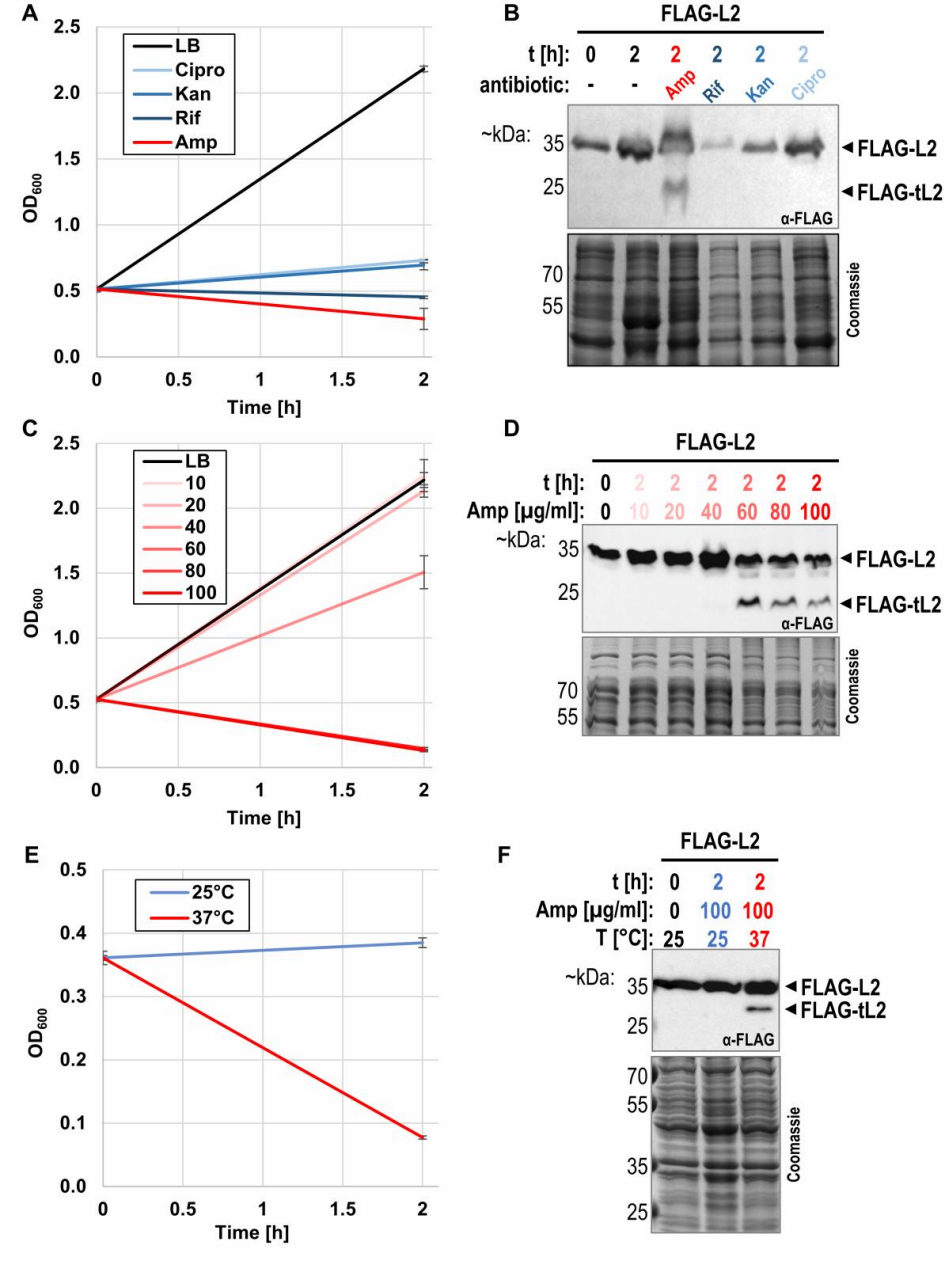
FIGURE 4: tL2 is only generated when cell lysis occurs. **(A)** Growth of strain BW25113 pZS*33-lacZa-FLAG-rplB was monitored by measuring optical density at 600 nm upon treatment for 2h with 10 µg/mL ciprofloxacin (Cipro), 25 µg/mL kanamycin (Kan), 75 µg/mL rifampicin (Rif), or 100 µg/mL ampicillin (Amp). As a control, unstressed cells were simultaneously grown in LB with no antibiotic added (n=3). Samples were taken for western blot analysis in **(B)** before and 2 hours after the addition of the antibiotics. **(C)** Growth of BW25113 pZS*33-lacZa-FLAG-rplB was monitored by measuring optical density at 600 nm upon treatment with 10-100 µg/mL ampicillin for two hours. Note that the curves for 60 µg/mL, 80 µg/mL, and 100 µg/mL ampicillin are overlapping. As a control, unstressed cells were simultaneously grown in LB only (n=3). Samples were taken for western blot analysis in **(D)** before and after the stress. **(E)** Growth of BW25113 pZS*33-lacZa-FLAG-rplB cells was monitored by measuring optical density at 600 nm before and after treatment with 100 µg/mL ampicillin (n=3). Cells were first grown in LB at 25°C and then split upon antibiotic addition into two cultures and further incubated either at 25°C (blue) or 37°C (red). Samples were taken for western blot analysis in **F** before the stress at OD_600 _~ 0.4 (t=0h) and 2 hours after the stress.

**Figure 5:**
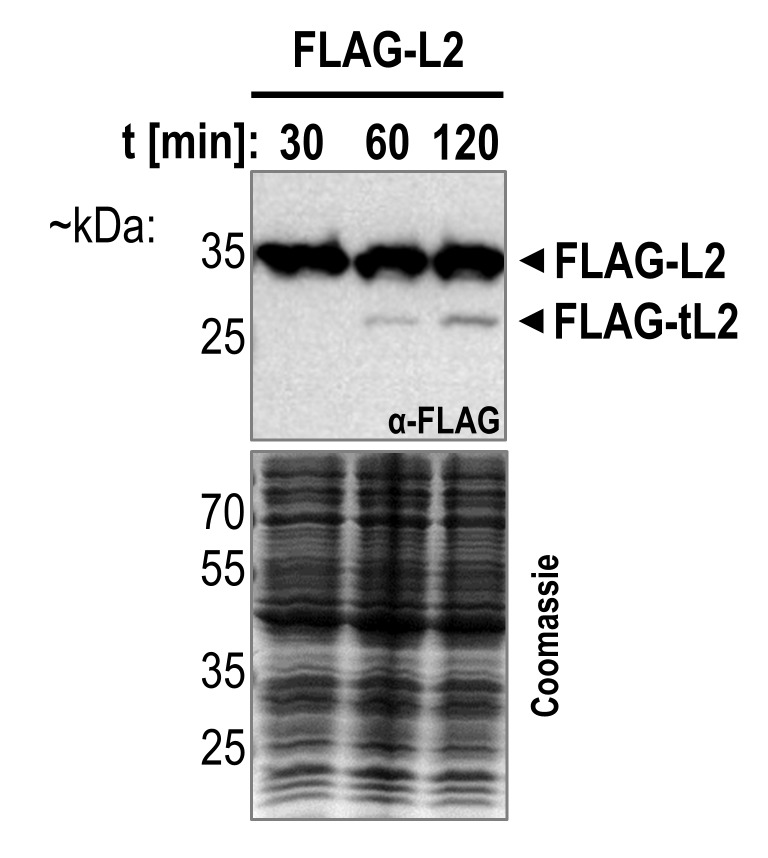
FIGURE 5: Incubation of cell extract from untreated cells leads to the production of tL2. Cell extract was prepared by mechanical opening of exponentially growing BW25113 pZS*33-lacZa-FLAG-rplB cells as is described for the sucrose gradient analysis in material and methods. Aliquots of the cell extract were incubated at 37°C for the indicated span of time, followed by western blot analysis using the anti-FLAG antibody and a Coomassie gel as loading control.

Despite the proposed inhibitory role of tL2 in DNA replication, Chodavarapu *et al*. did not observe a growth phenotype when levels of tL2 were artificially increased *in vivo* 37. This agrees with our own findings, as we equally recorded no growth phenotype when overexpressing a truncated *rplB* gene encoding tL2 (data not shown). The authors argued that this does not necessarily indicate that tL2 does not inhibit DNA replication in *E. coli* cells, but that other interconnected factors may be required, and that the conditions upon which all necessary regulatory proteins are present have simply not been identified. We therefore investigated whether tL2 is produced under different growth and stress conditions besides ampicillin treatment. DNA replication has been shown to be inhibited by a plethora of factors during stationary phase 50,51 and the number of persister cells increased in later stationary phase 24. Thus, we first sampled cells expressing the *FLAG-rplB *gene throughout different stages of exponential growth and into extended stationary phase. Contrary to previous findings 37, we did not detect tL2 in the exponential growth phase or after 2 weeks in stationary phase (Figure S3), indicating that tL2 is not present for DNA replication regulation in neither nutrient rich nor nutrient depleted conditions. Next, we investigated tL2 production upon antibiotic treatments other than ampicillin. As shown in **Figure 4A** and **B**, no tL2 was detected after the addition of RNA polymerase inhibiting rifampicin, 30S targeting kanamycin, or topoisomerase IV inhibiting ciprofloxacin. Since tL2 production appeared to be ampicillin specific, we further scrutinized tL2 production under different ampicillin concentrations. As shown in **Figure 4C** and **D**, tL2 is only detected at ampicillin concentrations that are sufficient for widespread cell lysis as evidenced by a stark decline in the optical density. Finally, since beta-lactam antibiotics are only effective when peptidoglycan synthesis is occurring, we expected a slower growth rate to result in a higher tolerance to the treatment. Therefore, we investigated tL2 production upon addition of 100 µg/mL ampicillin at both 37°C and 25°C. As expected, the decreased growth rate at 25°C led to a growth arrest after the addition of ampicillin but not to the widespread cell lysis as was observed when the culture was shifted to 37°C (**Figure 4E**). In agreement with our other findings, tL2 was only detected at 37°C (**Figure 4F**). Taken together, these results indicate that tL2 is only produced when cell lysis occurs. To investigate whether ampicillin treatment is nonetheless required for tL2 production or whether tL2 is similarly generated when untreated cells are lysed, we prepared cell extract from unstressed *E. coli* cultures by mechanical opening of the cells. The cell extract was then further incubated at 37°C, which led to a gradual increase of tL2 levels (**Figure 5**), indicating that prior ampicillin treatment is not required for tL2 synthesis.

### tL2 is not found in persister cells

**Figure 6:**
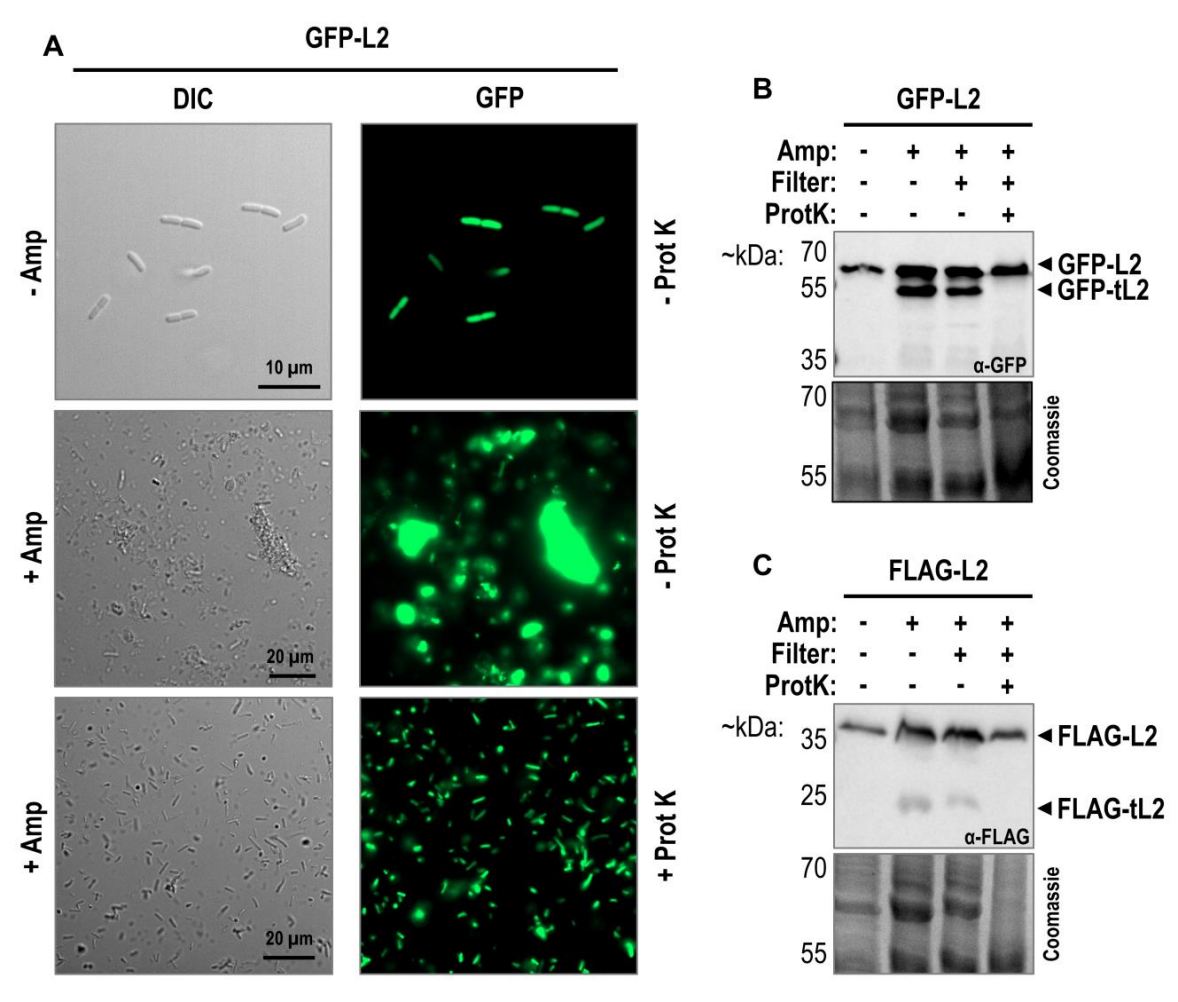
FIGURE 6: tL2 is not found in intact persister cells after ampicillin treatment. **(A)** Exponentially growing BW25113 pZS*33-lacZa-GFP-rplB cells were treated for 2 hours with 100 µg/mL ampicillin and intact persister cells were isolated as described in the main text without (*middle*) or with (*bottom*) the addition of proteinase K. Untreated (*top*) and differentially purified persister cells were visualized using differential interference contrast (DIC) and fluorescence microscopy. Samples were taken for western blot analysis in **(B)** during the different purification steps for intact persister cells isolation. The used primary antibody is indicated inside of the western blot box and a Coomassie gel was used as a loading control. tL2 is still detected after the ampicillin treatment (Amp) and when filtered through a paper filter by gravitation but disappears upon treatment with proteinase K (ProtK). **(C)** Western blot equal to B, but BW25113 pZS*33-lacZa-FLAG-rplB persister cells were isolated.

As we previously observed tL2 production exclusively upon lytic conditions and independent of ampicillin addition, we speculated that the generation of tL2 does not occur in the cytosol of surviving persister cells but is merely an artifact of cell disruptions. To scrutinize our assumption, we employed a GFP-L2 fusion protein to directly monitor the r-protein inside of intact cells by fluorescence microscopy. It should be noted, that r-protein synthesis is generally tightly regulated and no or only minor free uL2 is found in the cytosol (see fractions Top 1-3 in Figure S4D). Incorporated into the fully assembled ribosome, arginine 214 of uL2 is not directly accessible for potential proteases (Figure S2). Thus, to ensure that any processing in the free protein pool, we first set out to confirm that GFP-L2 is still incorporated into the large subunit. Sucrose gradient analysis of cell lysates containing FLAG-L2, GFP-L2, or free GFP shows that GFP-L2 clearly is incorporated into 50S subunits and to a lesser extend into assembled 70S ribosomes (Figure S4C and D). Furthermore, western blot analysis showed that neither FLAG-L2 nor GFP-L2 were found in the top fractions, indicating that the fusion proteins were efficiently assembled into the 50S subunit (Figure S4D and Figure S5). Having confirmed that the localization and incorporation of GFP-L2 in ribosomes is comparable to the native uL2 proteins, we continued with the purification of intact persister cells. In their pioneering work, Keren *et al*. performed a single centrifugation step to isolate non-lysed cells after ampicillin treatment 39. Later studies often included additional purification steps, such as serial washing of the cell pellet with phosphate-buffered saline (PBS) 25. To ensure the isolation of pure persister cells, we slightly adapted the latter protocol and included an initial filtra-tion step to remove bulky aggregates of cell debris. However, as shown in **Figure 6A**, differential interference contrast (DIC) microscopy revealed large amounts of residual cell debris*. *Furthermore, fluorescence microscopy indicated that the accumulated cell debris contained abundant GFP-L2 or GFP-tL2 fusion proteins. We therefore included an additional proteinase K treatment step to digest extracellular proteins before washing of the surviving persister cells with PBS. As the bottom of **Figure 6A** indicates, removal of the cell debris was complete and a clear* GFP-*signal was detected inside of the isolated intact persister cells. Moreover, western blot analysis in **Figure 6B** clearly shows that all the fluorescence signal originates solely from full-length GFP-L2 fusion proteins, as the band corresponding to the truncated GFP-L2 variant was no longer observed. The same observation was true when the procedure was repeated with cells containing the FLAG-L2 fusion protein (**Figure 6C**). We therefore concluded that the observed truncation of uL2 upon ampicillin treatment must be attributed to proteolysis after lysis of the cells and that tL2 is not produced intracellularly of ampicillin tolerant persister cells to downregulate DNA replication.

## DISCUSSION

### tL2 is an artifact of cell lysis

Ribosomal proteins play an integral role in both ribosome structure and function. Independently of this core function, previous studies have identified an ever-increasing number of extraribosomal, moonlighting activities for numerous r-proteins (reviewed in 28,29). Moreover, the degradation of ribosomal complexes was shown to contribute to translational suppression in persister cells 25. We were therefore intrigued by the possibility that the newly freed r-proteins further contribute to persister cell survival by previously undescribed mechanisms. As the bacteriolytic properties of beta-lactam antibiotics have routinely been used to study persister cells, we treated *E. coli* cells with ampicillin to separate tolerant persister cells from the treatment sensitive cells as previously described 39. During the subsequent analysis of the r-protein content of the surviving persisters, we reproducibly observed stable truncation products of the investigated r-proteins (**Figure 1**). Promisingly, we identified a truncation of the large subunit protein uL2 lacking 59 amino acids from its C-terminus (tL2, **Figure 2**). Previously, Chodavarapu et al. described tL2 as a regulator of DnaA activity *in vitro* 37. We therefore speculated that *in vivo*, tL2 inhibits DNA replication in ampicillin induced persister cells to arrest the cell division machinery and thereby neutralize the lytic properties of the beta-lactam antibiotic. In line with this hypothesis, but contrary to the findings of Chodavarapu et al., we did not observe tL2 in unstressed, exponentially growing *E. coli* cells (see **Figure 2**, **Figure 3**, **Figure 4**, and **Figure 6**). Furthermore, tL2 was gradually produced upon addition of the antibiotic and vanished when the persister cells were recovering (**Figure 3**). However, we observed tL2 production only upon ampicillin treatment with doses above the minimal inhibitory concentration (MIC) that resulted in widespread cell lysis (**Figure 2**, **Figure 3**, and **Figure 4**) and not with concentrations below the MIC, where no or only minimal cell lysis was observed (**Figure 4C** and **D**). Additionally, tL2 was not generated upon treatment with other non-lytic antibiotics (**Figure 4A** and **B**). We thus speculated that the observed generation of tL2 is an artifact of cell lysis. Consistent with this assumption, we observed the gradual production of tL2 in cell extract isolated from untreated, non-persister cells when incubated at 37°C *in vitro* (**Figure 4E** and **F**). Finally, employing a GFP-L2 fusion protein, we showed by combining fluorescence microscopy with western blot analysis that no tL2 is found intracellularly of ampicillin induced persister cells (**Figure 6**). We therefore concluded that the generation of tL2 upon ampicillin treatment must solely be attributed to proteolysis of uL2 proteins released from disrupted cells. Importantly, these findings do not exclude the possibility of other environmental conditions that could lead to the generation of intracellular tL2. However, these findings indicate that the responsible protease for uL2 cleavage is physically separated from the r-protein in unstressed *E. coli* cells. Hence, we tested a series of diversely localized proteases using gene deletion strains from the Keio collection 52. Unfortunately, we did not identify a single responsible serine protease for uL2 processing (Figure S6).

### Proteinase K treatment improves persister cells isolation for downstream investigations

For our initial screening experiments to study the r-protein content in ampicillin tolerant persister cells, we isolated the surviving cells after antibiotic treatment with a single centrifugation step as previously described 39. However, microscopic imaging revealed that a single centrifugation step was insufficient for separating intact persister cells from accumulated cell debris (data not shown). We therefore first adapted a previously published protocol 25*, *which additionally purified the persister cells by employing several washing steps with PBS, to further include an initial filtration step to remove larger aggregates of cell debris. However, the combination of DIC and fluorescence microscopy imaging revealed that this protocol was still insufficient to remove all residual cell debris (**Figure 6A**, middle). We therefore further adapted the protocol by digesting extracellular proteins with proteinase K as previously described 53*. *As shown at the bottom of **Figure 6A**, this approach allowed the efficient removal of cell debris to obtain pure, intact cells. As described above, after this careful isolation, we did not detect tL2 intracellularly of intact persister cells*. *We therefore finally repeated the western blot analysis for several of the initially tested r-proteins in **Figure 1** using samples collected *i)* from unstressed cells, *ii)* directly after ampicillin treatment, or *iii)* from purified persister cells as described above. As Figure S7 shows, the same stable processing products are present in the ampicillin treatment sample as previously observed (**Figure 1**) but are often not or only minorly detected in the intact persister cells. Thus, *t*hese results further highlight the importance of including additional purification steps for downstream applications and analyses of persister cells since repeated washing with buffer is not sufficient to remove cell debris contaminants and therein contained potential artifacts*. *Our finding is underscored by the study of Cho et al. performed in *E. coli*, which revealed a discrepancy between the protein composition of persister ribosomes isolated from cell lysate fractionated on sucrose gradients compared to affinity purified ribosomes 25. The authors speculated that the degraded ribosomes in the sucrose gradient may have originated from lysed non-persister cells but dismissed this possibility as unlikely, since previous repetitive washing of the surviving persister cells supposedly removed the residual cell debris. However, our results indicate that the cell debris likely was not completely removed and therein contained degraded ribosomes contaminated the sucrose gradient analysis. Thus, our results therefore invite for the careful re-examination of previously published studies that relied on potentially insufficient protocols for persister cell purification.

## MATERIAL AND METHODS

### Bacterial strains and growth conditions 

If not indicated otherwise, wildtype *E. coli* K-12 strain BW25113 52,54 and its derivatives were used in this study. For more detailed information on employed bacterial strains and plasmids, see Table S1 and Table S2. Except where otherwise specified, bacterial cultures were grown in LB medium (10 g/L peptone, 5 g/L yeast extract, 10 g/L NaCl) at 37ºC while shaking at 165 rpm. If required for plasmid retention, 30 µg/mL chloramphenicol was added to the medium. For antibiotic treatments, 100 µg/mL ampicillin, 75 µg/mL rifampicin, 25 µg/mL kanamycin, or 10 µg/mL ciprofloxacin were supplemented to the culture in early exponential phase. For ampicillin recovery experiments, the culture was centrifuged at 3,000*g for 8 minutes, washed with 20 mL of 1x PBS (137 mM NaCl, 2.7 mM KCl, 10 mM Na_2_HPO_4_, 1.8 mM KH_2_PO_4_, pH 6.8) and finally resuspended in fresh LB medium.

### Plasmid construction

Detailed information on all primers and plasmids used in this study is provided in Table S2. In general, genes of interest were amplified by PCR (Thermo Scientific, 2x Phusion Master Mix with HF Buffer) from genomic DNA (gDNA) and subsequently digested with appropriate restriction enzymes using the FastDigest restriction enzymes system from Thermo Scientific. The digested gene fragments were ligated (Thermo Scientific, T4 DNA Ligase) into the corresponding, equally digested vectors. Point mutations, various tags, and deletions were introduced by inverse PCR followed by blunt-end or sticky-end ligation. For simulated endogenous expression and in order to facilitate genetic manipulation of the essential *rplB* gene, the corresponding part of the larger *S10* operon was cloned into a low-copy plasmid under leaky P*_lac_* promoter expression (55, Figure S1C). To retain as many potential regulatory elements of *rplB* expression as possible, the last 30 bp of the upstream *rplW* gene were included and fused in-frame with the first 180 bp of the *lacZ* gene. Thus, both the endogenous ribosome binding site and any potential co-translational regulatory effects were conserved (Figure S1B).

### Western blot analysis 

At the indicated time points, OD_600_ of the bacterial culture was measured and samples were harvested at 10,000*g for 1 min at room temperature. 2x Laemmli buffer (4% SDS, 20% glycerol, 0.004% bromophenol blue, 125 mM Tris base, 10% beta-mercaptoethanol, pH 6.8) was added to the pellet to a final calculated concentration of 20 OD units per mL. Cells were opened and proteins were denatured simultaneously by cooking the resuspended pellet at 85°C for 5 minutes. Generally, 0.1 OD units were loaded per sample and proteins were separated on 10% or 12.5% SDS polyacrylamide gels. If necessary for the analysis of lower molecular weight proteins, 16% tris-tricine gels were used. Separated proteins were wet transferred to 0.2 µm nitrocellulose membranes (Amersham Protran) at 100 V for 1 h at 4 ºC in 1x transfer buffer (25 mM Tris base, 192 mM Glycine, pH 8.3). The membrane was first blocked using 5% milk powder in 1x PBS supplemented with 1% Tween-20 (1x PBS-T) and then probed with anti-FLAG (antibodies-online GmbH ABIN99294, raised in rabbit), anti-His (Cytiva 27471001, raised in rabbit), or anti-GFP (Proteintech 66002-1-Ig, raised in mouse) antibodies. For r-proteins, lab stock of primary antibodies raised in goat was employed. Anti-rabbit IgG (Cell Signaling Technology 7074S), anti-goat IgG (Sigma Aldrich A5420) or anti-mouse IgG (Proteintech RGAM001) each coupled to horseradish peroxidase was used as a secondary antibody. Blots were developed using the SuperSignal West Pico PLUS Kit chemiluminescent reagent from Thermo Scientific and the BioRad ChemiDoc Imaging system was used for signal detection.

### GFP-L2 incorporation into the ribosome by sucrose gradients centrifugation

To determine if the GFP-L2 fusion protein was still incorporated into the ribosome, cell extracts were separated on sucrose gradients followed by western blot analysis. First, 130 mL of exponentially growing cells were poured over 40 mL frozen LB containing 30 µg/mL chloramphenicol and harvested at 10,000*g for 10 min at 4 °C. The resulting cell pellet was washed with 10 mL of cold 1x PBS at 10,000*g for 3 min at 4 °C. The final pellet was resuspended in 1.5 mL of cold 1x TMN buffer (10 mM Tris, 10 mM MgCl_2_, 50 mM NH_4_Cl, 6 mM beta-mercaptoethanol, pH 7.6), and cells were lysed using a OneShot cell disruptor (Constant Systems Ltd.) at 1.4 kbar. Cell debris was removed by centrifugation at 30,000*g for 15 min at 4 °C. Of the collected supernatant, 20 A_260_ units were loaded onto a 15-35% sucrose gradient in 1x TMN buffer and centrifuged at 23,000 rpm for 15 h at 4 °C using a SW32 Ti rotor. Following centrifugation, the gradient was fractionated using a piston gradient fractionator (Biocomp Instruments), collecting 22 fractions of 1.5 mL each. Simultaneously, the absorbance was measured at 260 nm while GFP fluorescence was excited at 470 nm and measured at 535 nm. Proteins in the fractions were precipitated by addition of 350 µL of 100% trichloroacetic acid (TCA) to each fraction followed by incubation on ice for 1 h. The precipitated proteins were collected at 18,000*g for 15 min at 4 °C and washed with 400 µL of acetone. Final pellets were air-dried and resuspended in 2x Laemmli buffer for western blot analysis.

### Persister cell isolation

Persister cells were isolated after ampicillin treatment by gravity filtration through a GE Whatman Folded Filter Paper (595 1/2; CAT No. 10311647) and subsequent centrifugation at 1,500*g for 10 min at 4 ºC. Cell debris was removed according to a previously established protocol 53. In short, the pellet was resuspended in protease buffer (50 mM Tris base, 7.5 mM CaCl_2_, pH 8.8), followed by the addition of 50 µg/mL Proteinase K (QIAGEN, Mat. No. 10194990) and incubated at room temperature for 30 min. Proteinase K was inactivated in the presence of 10 mM PMSF for 10 min. Next, intact cells were pelleted at 1,500*g for 3 min at 4ºC. The pellet was washed three times in 1 mL of 1x PBS and the final pellet was resuspended in 10-200 µL of 1x PBS, adjusted according to pellet size. A 3 µL aliquot was taken for fluorescence microscopy, while the remainder was subsequently analyzed by western blot.

### Microscopic images

Microscopy samples were directly loaded on VWR microscope slides and fixed with cover slides. Fluorescence and DIC images were captured using a Zeiss Axio Imager Z2 upright microscope (Carl Zeiss Microscopy GmbH, Germany) equipped with a Plan-Apochromat 63x/1.4 Oil DIC M27 objective. The Zeiss filter set 38 for eGFP (BP 470/40, FT 495, BP 525/50) was employed to capture fluorescence signals under consistent exposure times (2000 ms). Images were captured using a Photometrics PRIME CMOS camera controlled via Zeiss ZEN blue 3.3 Pro software. Finally, images were processed using ImageJ/FIJI software (National Institutes of Health, USA).

## AUTHOR CONTRIBUTION

MF: Conceptualization, Methodology, Investigation, Writing – Origi-nal Draft, Visualization, Supervision, Funding acquisition HC: Valida-tion, Investigation, Writing – Review & Editing IM: Conceptualization, Resources, Data Curation, Writing Review and Editing, Project Ad-ministration, Funding acquisition.

## CONFLICT OF INTEREST

The authors declare no conflicts of interest.

## SUPPLEMENTAL MATERIAL

Click here for supplemental data file.

All supplemental data for this article are available online at www.microbialcell.com/researcharticles/2025a-fasnacht-microbial-cell/.
