# Supplementary material for: Ampicillin treatment in persister cell studies may cause non-physiological artifacts
Source: Microb Cell. 2025 Mar 20;12:53–64. doi: 10.15698/mic2025.03.845 (PMC12039935; doi:10.15698/mic2025.03.845)
Supplement: Supplementary file 1 [file mic-12-053-s01.pdf]

Supplemental Data

Ampicillin treatment in persister cell studies may cause non-physiological artifacts

Michel Fasnacht[*, a, b], Hena Comic[a, b], Isabella Moll[*, a, b]

a Max Perutz Labs, Vienna Biocenter Campus (VBC), Dr.-Bohr-Gasse 9 / Vienna Biocenter 5, 1030, Vienna, Austria.
b University of Vienna, Max Perutz Labs, Department of Microbiology, Immunobiology and Genetics Dr.-Bohr-Gasse 9 / Vienna Biocenter 5, 1030, Vienna, Austria.

*corresponding authors
michel.fasnacht@univie.ac.at, Dr.-Bohr-Gasse 9, 1030 Vienna, Austria
isabella.moll@univie.ac.at, Dr.-Bohr-Gasse 9, 1030 Vienna, Austria

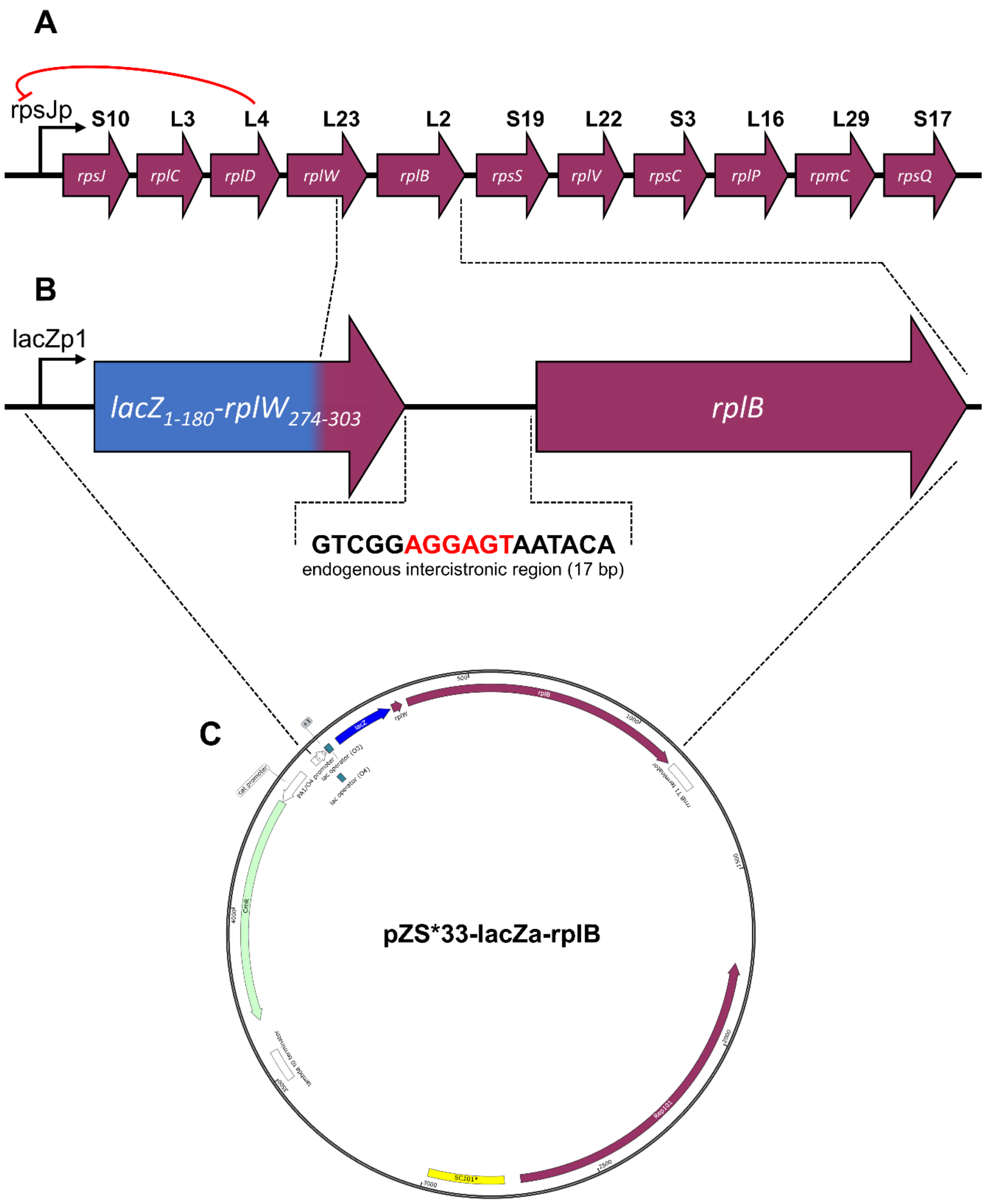

Figure S1. Plasmid construction for simulated endogenous expression of *rplB*

A Not-to-scale representation of the genomic region encompassing the *S10* operon. Genes are represented as arrows with the corresponding r-protein marked above. As described in the main text, uL4 has been shown to inhibit the transcription of the operon in a negative feedback loop. The corresponding region encoding for *rplB* and partially for the upstream *rplW* gene that was fused to the first 180 bp of the *lacZ* gene is enlarged in **B**. Furthermore, the complete intercistronic region is shown with the Shine-Dalgarno sequence highlighted in red. **C** Complete plasmid map of the final construct (generated in SnapGene).

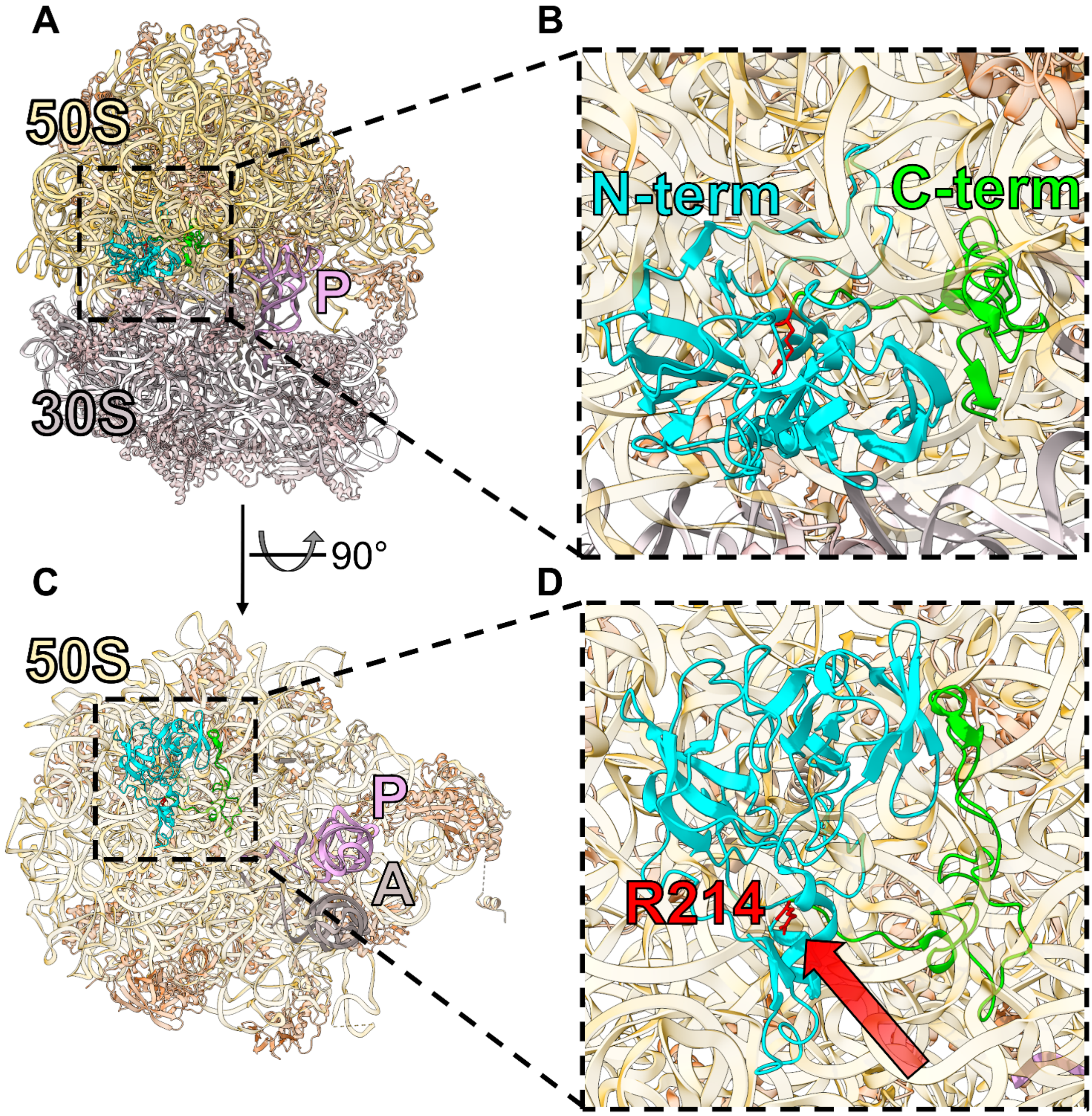

Figure S2. Arginine 214 of uL2 is not accessible for proteolytic cleavage when incorporated in the ribosome

A High-resolution cryo-EM structure of the *E. coli* 70S ribosome with the r-protein uL2 highlighted and the corresponding region enlarged in **B**. The last 59 C-terminal amino acids of uL2 are marked in green, whereas the remaining N-terminal part after cleavage (tL2) is shown in cyan. Small (30S) and large (50S) subunits of the assembled ribosome are indicated, as well as P-site (P) and A-site (A) tRNAs. **C&D** Same as in **A&B**, but with the 30S subunit removed and the 50S subunit rotated by 90° as indicated. Furthermore, the cleavage site at arginine 214 (R214) is highlighted in red. The structure was generated in ChimeraX from PDB entry 7K00 [56].

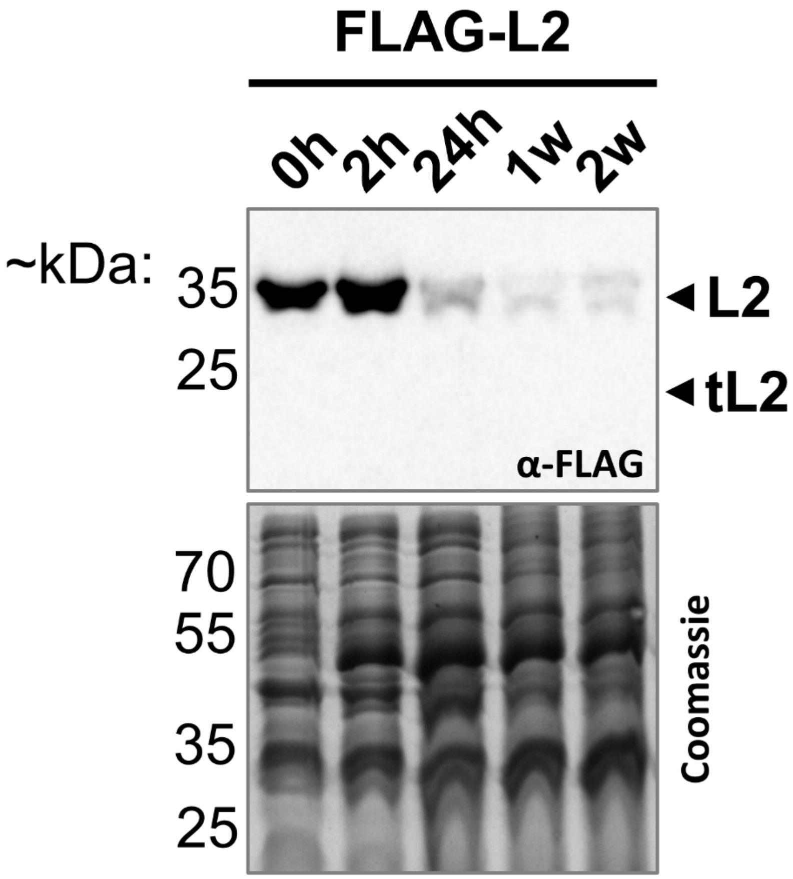

Figure S3. tL2 is neither found in exponential nor in long-term stationary phase

BW25113 pZS*33-lacZa-FLAG-rplB cells were grown in LB at 37°C into extended stationary phase and samples were taken at the indicated time points for western blot analysis. Shown is a representative western blot with the used primary antibodies indicated inside of the western blot box. A Coomassie gel was used as a loading control. No tL2 is detected after 2 weeks (2w) of growth. Consistent with previously published results [23], the signal corresponding to the full-length uL2 is reduced upon prolonged incubation time.

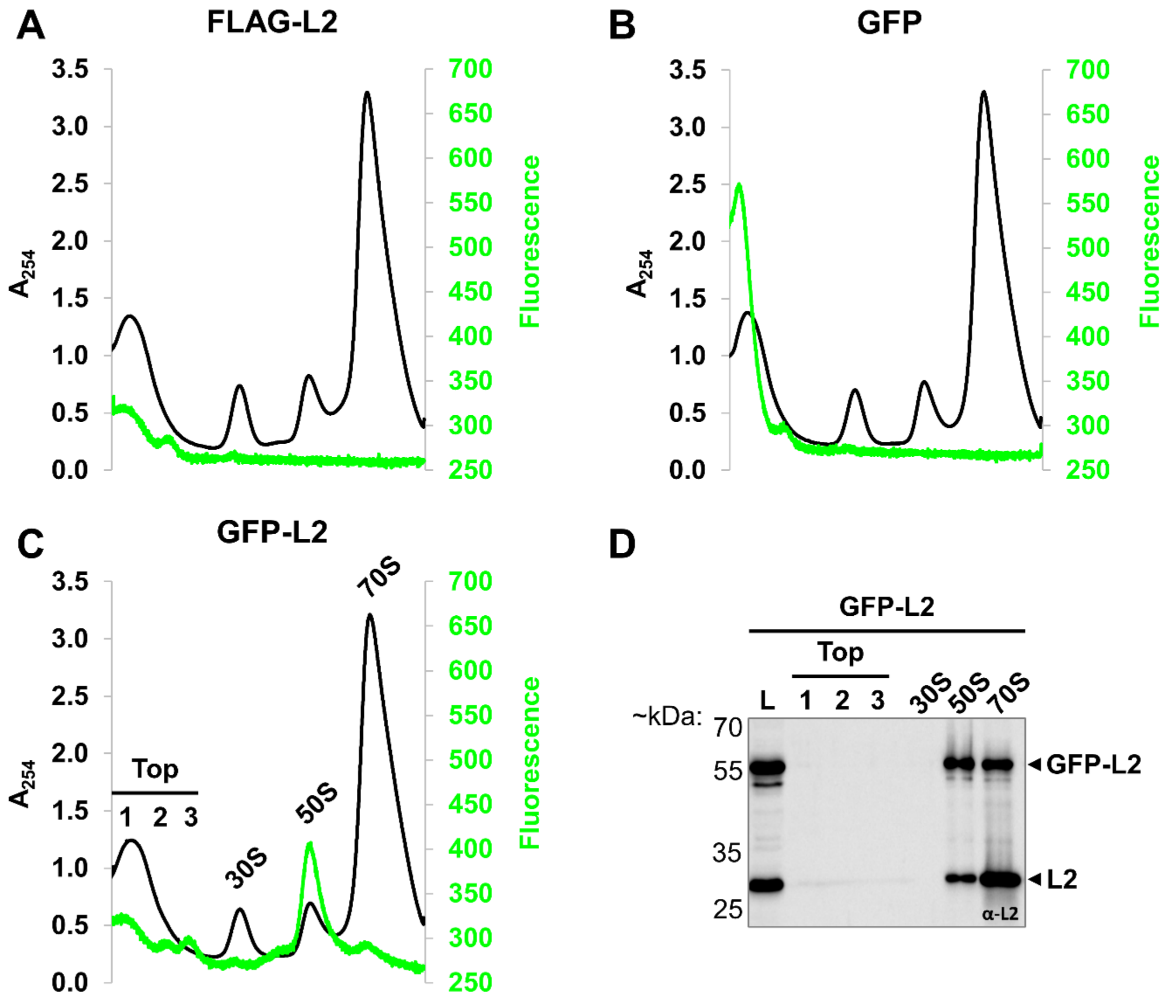

Figure S4. GFP-L2 is incorporated into the large ribosomal subunit

Cell extracts from exponentially growing wildtype BW25113 cells transformed with **A** pZS*33-lacZa-FLAG-rplB, **B** pZS*33-lacZa-GFP, or **C** pZS*33-lacZa-GFP-rplB were separated on 15-35% sucrose gradients. Elution of ribosomal particles was monitored (A_{254}) simultaneously with the GFP-fluorescence signal. **D** Western blot analysis with the used primary antibody indicated in the box. TCA precipitated proteins from the indicated fractions in **C** were loaded together with non-fractionated cell lysate (L). N-terminally GFP-tagged L2 (GFP-L2) is detected in both the free 50S subunit and the completely assembled 70S ribosomes.

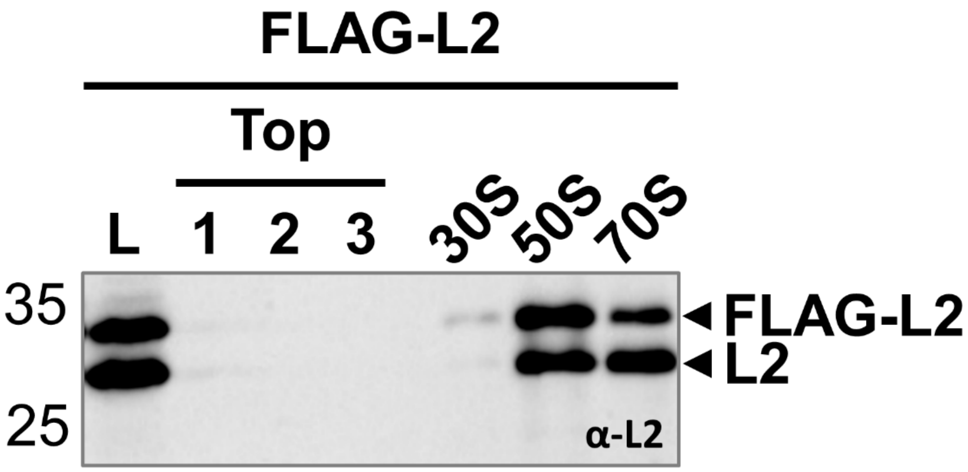

Figure S5. FLAG-L2 is incorporated into the large ribosomal subunit
Western blot analysis with the used primary antibodies indicated in the box. Equally to Figure S4C&D, TCA precipitated proteins from the collected fractions in Figure S4A were loaded together with non-fractionated cell lysate (L). N-terminally FLAG-tagged L2 (FLAG-L2) is detected in both the free 50S subunit and the completely assembled 70S ribosomes.

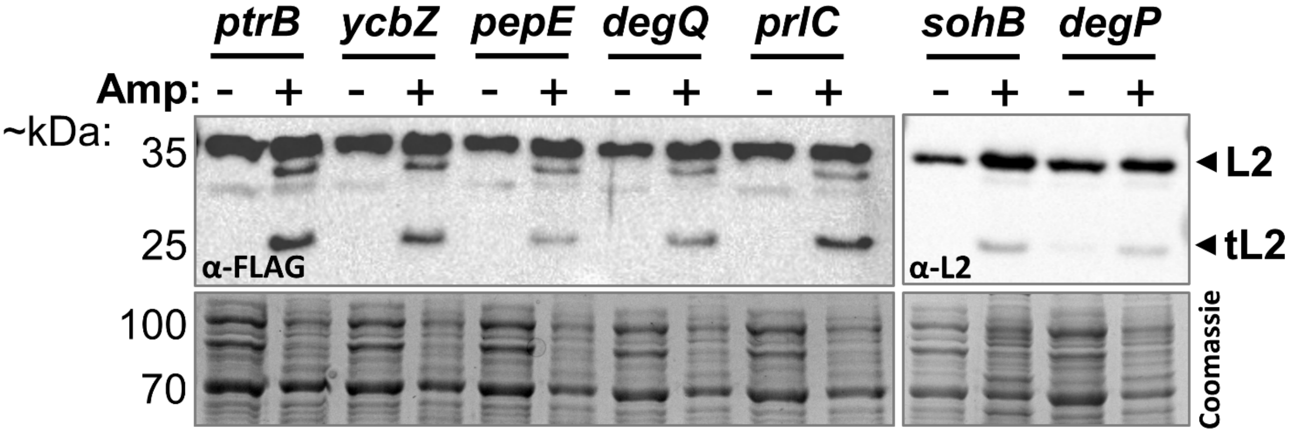

Figure S6. No single protease responsible for the generation of tL2 has been identified

Western blot analysis of samples taken from different Keio collection strains [52] with the indicated single genes deleted before and after treatment with 100 µg/ml ampicillin (Amp) for 2 hours. Strains on the left were transformed with plasmid pZS*33-lacZa-FLAG-rplB, whereas the strains on the right were not. Accordingly, the used primary antibodies are indicated inside of the western blot boxes and Coomassie gels were used as loading controls. As shown, tL2 can still be detected in all strains, indicating that none of the corresponding proteases are uniquely responsible for the cleavage of uL2.

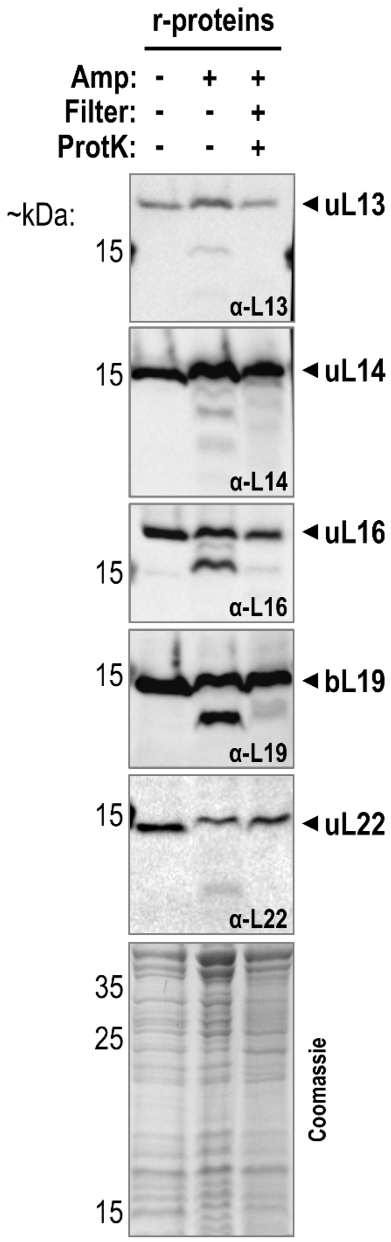

Figure S7. Most observed r-protein truncations are not found in purified persister cells

Western blot analysis of samples taken from wildtype *E. coli* strain BW25113 during different purification steps for intact persister cells isolation upon ampicillin treatment. The used primary antibodies are indicated inside of the western blot boxes and a Coomassie gel was used as a loading control. Comparable to Figure 1, truncations of several r-proteins are still detected after treatment with 100 µg/ml ampicillin for 2 hours (Amp). However, most of the investigated truncations disappear upon filtration and treatment with proteinase K (ProtK).

References

23. Reier K, Lahtvee P-J, Liiv A, and Remme J (2022). A Conundrum of r-Protein Stability: Unbalanced Stoichiometry of r-Proteins during Stationary Phase in Escherichia coli. mBio. 13(5): e01873-22. doi: 10.1128/mbio.01873-22.

52. Baba T, Ara T, Hasegawa M, Takai Y, Okumura Y, Baba M, Datsenko KA, Tomita M, Wanner BL, and Mori H (2006). Construction of Escherichia coli K-12 in-frame, single-gene knockout mutants: the Keio collection. Mol Syst Biol. 2: 2006.0008. doi: 10.1038/msb4100050.

56. Watson ZL, Ward FR, Méheust R, Ad O, Schepartz A, Banfield JF, and Cate JHD (2020). Structure of the bacterial ribosome at 2 Å resolution. eLife. 9: 1–62. doi: 10.7554/ELIFE.60482.
